# Socioeconomic status and its relation with breast cancer recurrence and survival in young women in the Netherlands

**DOI:** 10.1016/j.canep.2022.102118

**Published:** 2022-02-05

**Authors:** Marissa C. van Maaren, Bernard Rachet, Gabe S. Sonke, Audrey Mauguen, Virginie Rondeau, Sabine Siesling, Aurélien Belot

**Affiliations:** a Department of Research and Development, Netherlands Comprehensive Cancer Organisation, Utrecht, The Netherlands; b Department of Health Technology and Services Research, Faculty of Behavioural, Management and Social Sciences, Technical Medical Centre, University of Twente, Enschede, The Netherlands; c Inequalities in Cancer Outcomes Network (ICON), Department of Non-Communicable Disease Epidemiology, Faculty of Epidemiology and Population Health, London School of Hygiene and Tropical Medicine, London, United Kingdom; d Department of Medical Oncology, Netherlands Cancer Institute, Amsterdam, The Netherlands; e Department of Epidemiology and Biostatistics, Memorial Sloan Kettering Cancer Center, New York, United States; f INSERM U1219, Biostatistics team, University of Bordeaux, Bordeaux, France

**Keywords:** Young breast cancer patients, Socioeconomic status, Mortality, Recurrence, Joint modelling

## Abstract

**Background:**

Associations between socioeconomic status (SES) and breast cancer survival are most pronounced in young patients. We further investigated the relation between SES, subsequent recurrent events and mortality in breast cancer patients < 40 years. Using detailed data on all recurrences that occur between date of diagnosis of the primary tumor and last observation, we provide a unique insight in the prognosis of young breast cancer patients according to SES.

**Methods:**

All women < 40 years diagnosed with primary operated stage I-III breast cancer in 2005 were selected from the nationwide population-based Netherlands Cancer Registry. Data on all recurrences within 10 years from primary tumor diagnosis were collected directly from patient files. Recurrence patterns and absolute risks of recurrence, contralateral breast cancer (CBC) and mortality – accounting for competing risks – were analysed according to SES. Relationships between SES, recurrence patterns and excess mortality were estimated using a multivariable joint model, wherein the association between recurrent events and excess mortality (expected mortality derived from the general population) was included.

**Results:**

We included 525 patients. The 10-year recurrence risk was lowest in high SES (18.1%), highest in low SES (29.8%). Death and CBC as first events were rare. In high, medium and low SES 13.2%, 15.3% and 19.1% died following a recurrence. Low SES patients had shorter median time intervals between diagnosis, first recurrence and 10-year mortality (2.6 and 2.7 years, respectively) compared to high SES (3.5 and 3.3 years, respectively). In multivariable joint modeling, high SES was significantly related to lower recurrence rates over 10-year follow-up, compared to low SES. A strong association between the recurrent event process and excess mortality was found.

**Conclusions:**

High SES is associated with lower recurrence risks, less subsequent events and better prognosis after recurrence over 10 years than low SES. Breast cancer risk factors, adjuvant treatment adherence and treatment of recurrence may possibly play a role in this association.

## 1. Introduction

Women diagnosed with early stage breast cancer have a higher mortality risk as compared to the general population, even years after their initial breast cancer diagnosis [1,2]. This is mainly caused by the occurrence of (late) recurrences (local, regional and distant), second primary cancers and late side effects of treatment [2]. Although breast cancer occurs more frequently in higher socioeconomic classes, it presents with more favorable characteristics as compared to lower socioeconomic classes [3]. Several studies have shown that, after correction for prognostic factors, high socioeconomic status (SES) is associated with better breast cancer survival in multiple continents and countries [4–10]. However, these associations are not consistent across subgroups [3]. A Swedish study showed that survival differences among socioeconomic classes were most pronounced in younger breast cancer patients (<40 years) [11]. A Norwegian group [12] studied stage-specific survival in young breast cancer patients over time and showed that survival improved for young women of high SES, but not for low SES. Suggested reasons were lifestyle, comorbidity, access to treatment or the opportunity or ability of patients to make informed treatment choices [12]. However, comorbidity is unlikely to play a large role in young women, and access to treatment is expected to be equal in these countries. To further explore these mortality differences, it is important to take into account recurrent events. As breast cancer survival is largely determined by distant metastases (DM) [13] (which in turn are influenced by the occurrence of local (LR) or regional recurrences (RR) [14]), the relation between SES, the entire recurrent event process (including all subsequent events) and mortality has to be adequately assessed. This has not been done before, as recurrence and mortality are usually modeled on their own, rather than as a joint process.

Here, we do not aim to prove the already established association between recurrences and excess mortality, but we aim to describe *how* both processes are related and how it differs between socioeconomic classes in breast cancer patients < 40 years. Using data on all consecutive recurrences, and by using a joint modelling framework including the correlation with excess mortality, we provide a unique insight in the prognosis of young breast cancer patients according to SES.

## 2. Methods

### 2.1. Design and population

In this population-based historic cohort study, data was extracted from the Netherlands Cancer Registry (NCR). Trained and dedicated data managers prospectively register data on patient-, tumor- and treatment-related characteristics of all newly diagnosed malignancies following a notification of the nationwide network and registry of histo- and cytopathology in the Netherlands (PALGA). For primary invasive breast cancers diagnosed in 2005, an active follow-up was conducted in which data on recurrences within 10 years from diagnosis were collected directly from patient files. This additional data collection was performed outside of this study, but is not part of the routine data collection in the NCR. However, this data collection was performed by the same trained datamanagers as who perform the routine data collection, who have broad experience in the collection of follow-up data as it is regularly part of additional projects that are registered in the NCR.

In this study, all women < 40 years with primary stage I-III breast cancer diagnosed in 2005 in the Netherlands were identified from the NCR. Included patients were treated with local surgery in a Dutch hospital and did not have synchronous breast cancer (second breast cancer diagnosed within ≥90 days of the first).

### 2.2. Outcomes and definitions

We investigated the relationship between SES, recurrences and excess mortality within 10 years from diagnosis. SES was based on scores assigned to the four numbers of the Dutch postal code, extracted from the Netherlands Institute for Social Research. The scores arise from a principal component analysis on mean household income, percentage of inhabitants with a low income, percentage of low educatedness and percentage of unemployment [15]. The scores were decoded into deciles, which were consequently classified as low (deciles 1,2,3), medium (deciles 4,5,6,7) and high (deciles 8,9,10) SES.

Recurrences comprise LR, RR and DM, which were defined according to consensus-based event definitions [16]. In case of concurrent recurrences (at the same date) we analysed the one with the worst prognosis (DM first, then RR, then LR). However, in multivariable analysis, we combined all recurrences because of the low incidence of especially LR and RRs, and because we aimed to model the entire process of subsequent recurrences. Contralateral breast cancer (CBC) was defined as breast cancer in the opposite breast diagnosed 90 days of the primary tumor. The excess mortality hazard is defined as the mortality hazard of the patient population divided by the mortality hazard of the general Dutch population (obtained from Statistics Netherlands (https://www.cbs.nl/en-gb)), matched by age, gender, calendar year. This excess mortality hazard can be interpreted as the mortality (in)directly linked to the cancer under study (breast cancer in our case). Follow-up times were defined as the time between definite surgery of the primary tumor and any subsequent event (any recurrence, CBC, and death), with the corresponding event type. Patients alive after 10 years were censored at 10 years.

### 2.3. Statistical analysis

Patient-, tumor-, treatment-, and event-related characteristics according to SES were summarized using descriptive statistics and compared using the Chi-squared test. We estimated the absolute risk of recurrence as first event within 10 years (cumulative incidence function) using the non-parametric Aalen-Johansen estimator (stcompet command in Stata), in which death and CBC (as first event) were considered to be competing events. The absolute recurrence risk at time *t* represents the probability of experiencing a recurrence by time *t* in the presence of the other competing events [17,18]. As the occurrence of CBC is extremely low in our study population (see results), and follow-up was collected for the first primary breast cancer only, we decided to only include CBC in the descriptive statistics in which we focused on the first occurring event. In the multivariable analysis in which we included all subsequent events, we censored patients who experienced an invasive CBC at the date of occurrence of this event. In situ breast cancer events were ignored (so not marked as an event) in multivariable analysis as they do not affect recurrence or mortality risks.

Thereafter, we used a joint modelling approach. As mortality highly depends on the occurrence of recurrent events, it creates an informative censoring by preventing the occurrence of subsequent recurrences [19]. This type of informative censoring may lead to biased estimates when analysing recurrent events [20]. Joint modelling frameworks take this correlation into account by including a random effect shared by the recurrent event process and the mortality event [20–23]. We used the model developed by Belot et al. [21], which is based on two submodels: a model for the recurrence hazard and a model for the excess mortality hazard. Baseline hazards of both outcomes were modeled using cubic B-splines with one interior knot located at the median of the event-time distribution (3.7 years for recurrence, 5.1 years for mortality). Cumulative baseline hazards were approximated using the Cavalieri-Simpson approximation [24]. The random effect shared between the two hazards was assumed to follow a normal distribution with mean 0 and variance *θ*, and we included a scale parameter *γ* which multiplies the random effect in the linear predictor of the excess mortality hazard. The full likelihood was approximated with the adaptive Gaussian quadrature with 15 quadrature points and the optimization was done using a quasi-Newton algorithm, as implemented in the SAS proc nlmixed. In the multivariable model, we considered potential confounders based on clinical foreknowledge and literature. As we lacked information on SES of the reference population we used to estimate expected mortality, but we assumed excess mortality estimates to be similar to overall mortality estimates, we additionally executed the joint modelling analyses using overall mortality instead of excess mortality as outcome to verify this (for this we did not need a reference population).

All statistical tests were two-sided. Descriptive statistics and competing risk analyses were performed in Stata version 16.1, joint modelling analyses were performed in SAS version 9.4.

## 3. Results

The original follow-up cohort consisted of all breast cancer patients with operated primary non-metastatic unilateral invasive breast cancer, diagnosed in 2005. For this study, we requested data of 615 patients < 40 years from this cohort. We excluded four male patients, 11 patients who turned out to have a pathological in situ tumor, one patient who had macroscopic residual tumor left after surgery, seven patients treated with lumpectomy without additional radiotherapy and 67 patients treated with neoadjuvant systemic therapy (as it was not administered in many patients in 2005 and would need separate analysis), ending up with a final study population of 525 patients of whom 178 (33.9%) were of low, 203 (38.7%) of medium and 144 (27.4%) of high SES. Four patients did not have complete 10-year follow-up due to for example emigration.

Patients of high SES were more often diagnosed with stage II breast cancer as compared to patients of low and medium SES, who more often had stage I. Patients of high SES more often received endocrine therapy (Table 1).

### 3.1. Recurrence patterns

**Fig. 1.**
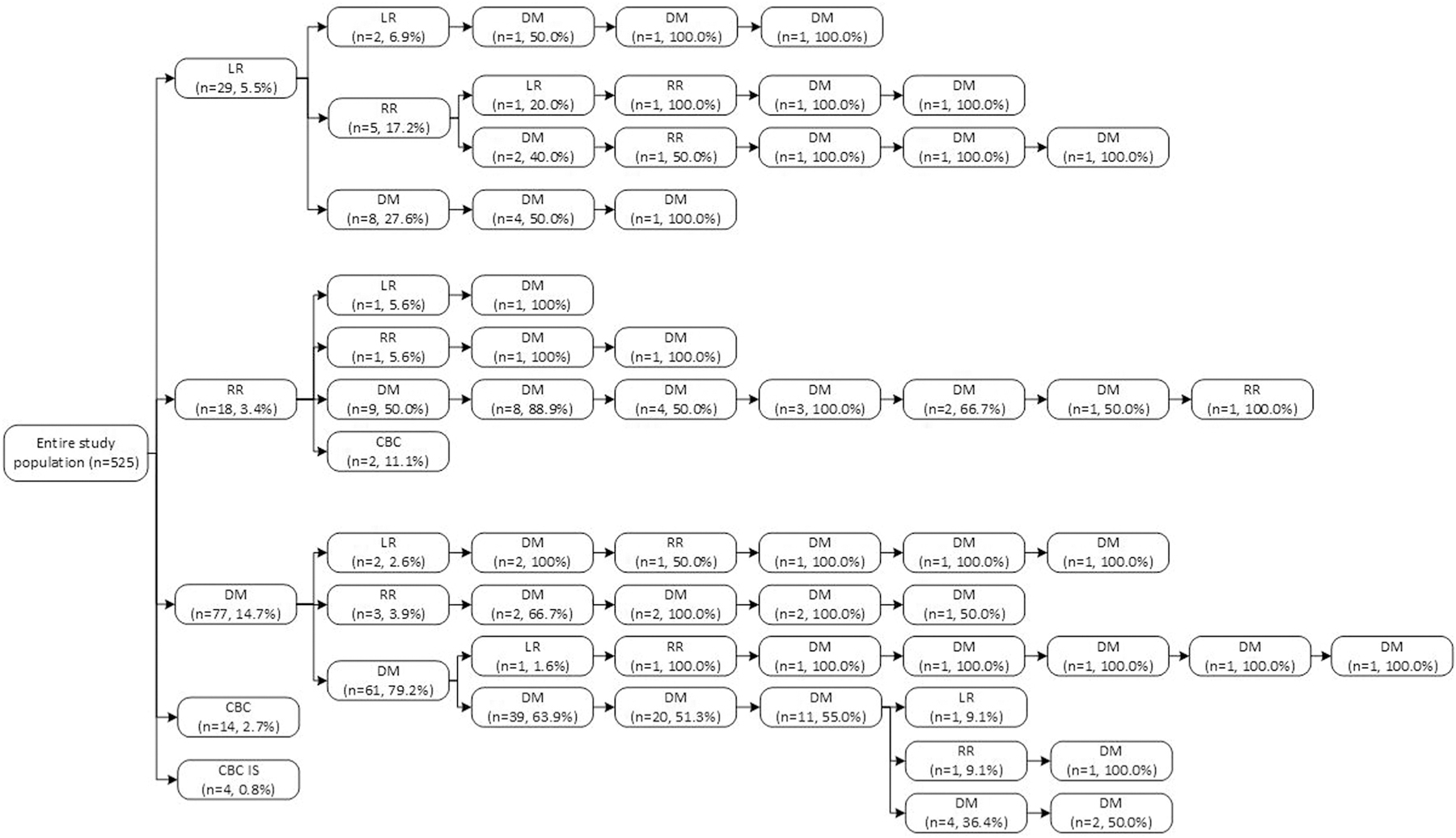
Patterns of recurrences in women < 40 years with primary non-metastatic breast cancer (n = 525). Abbreviations: LR = local recurrence, RR = RR, DM = distant metastasis, CBC = CBC, IS = In situ.

LR, RR and DM as first event were diagnosed in 5.5%, 3.4% and 14.7% of the complete study population within 10 years, respectively (Fig. 1). A contralateral invasive breast cancer event was diagnosed in 2.7% of the patients, while a contralateral in situ event was diagnosed in 0.8% of the patients. A considerable percentage of patients who experienced any event, developed a subsequent event. Of all patients who developed a LR as first event, 6.9% developed a subsequent LR, 17.2% developed a RR and 27.6% developed a DM as second event. Of all patients diagnosed with a RR as first event, 5.6% developed a LR, 5.6% a RR and 50.0% a DM as second event. Of all patients diagnosed with DM as first event, 2.6% were diagnosed with a LR, 2.9% with a RR and 79.2% with a subsequent DM as second event. The maximum number of consecutive events was nine. The recurrence hazard was highest around two years following diagnosis, with the highest hazard in patients of low SES (Fig. A1). Patients of high SES have the least subsequent recurrences, compared to low and medium SES (Figs. A2–A4).

### 3.2. First events according to SES

Median times to first recurrence were 2.6 years (IQR:1.6–4.1 years), 3.9 years (IQR:2.1–5.6 years) and 3.5 years (IQR:2.0–6.2 years) for low, medium and high SES, respectively. Table 2 shows the numbers and types of the first event (left panel) and of any events (right panel), stratified for SES. In patients with low SES recurrences occurred more often than in medium or high SES (29.8%, 22.2% and 18.1%, respectively), mostly distant metastases. CBC was rare in all groups. Overall, 90 patients (17.1% of total) died within 10 years: 37 (20.8%), 34 (16.8%) and 19 (13.2%) patients died in the low, medium and high socioeconomic group, respectively. Six patients died without experiencing any event: three (1.7%) of low, three (1.5%) of medium SES.

**Fig. 2.**
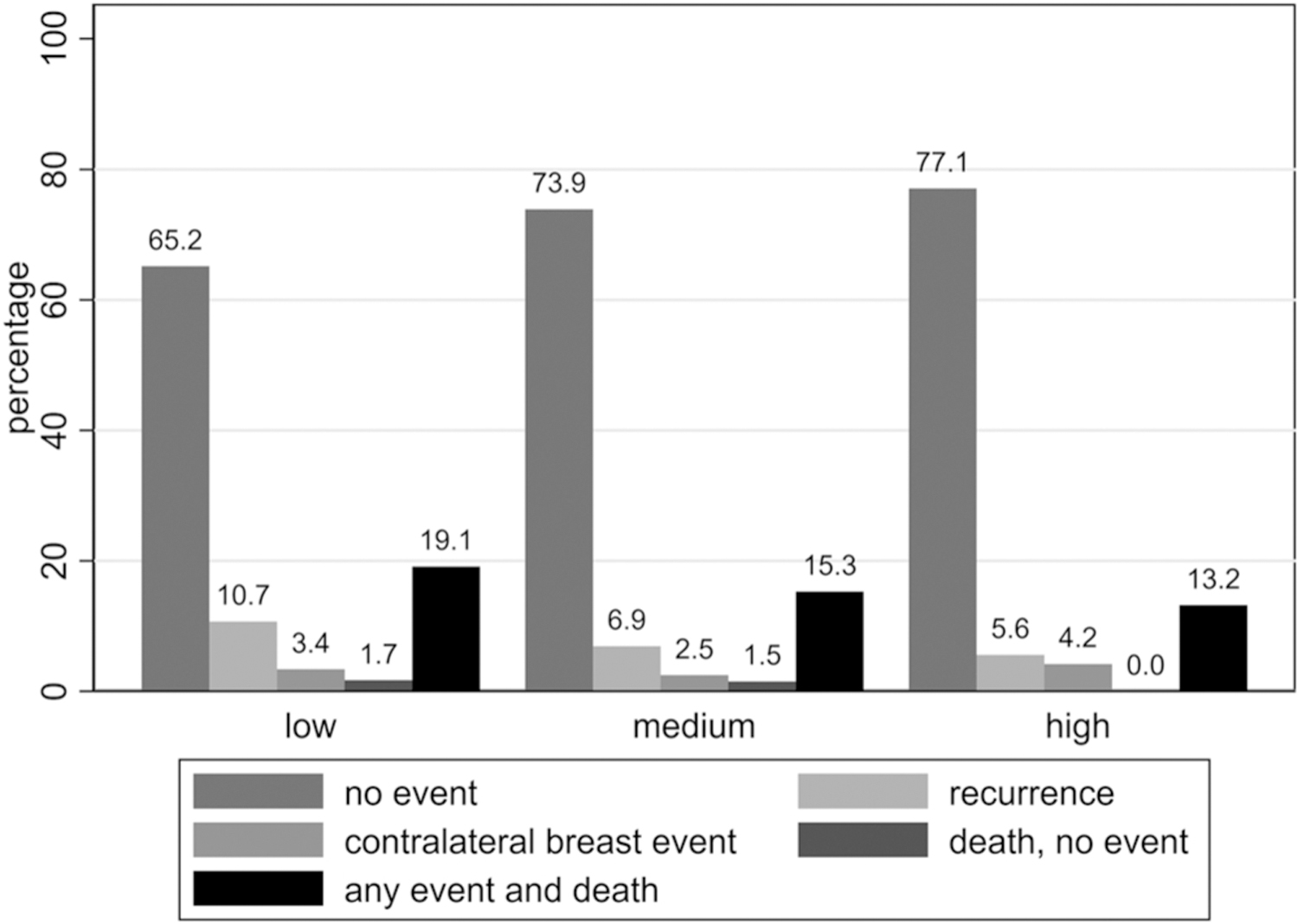
Percentages of (combinations of) events according to socioeconomic status in women < 40 years with primary non-metastatic breast cancer (n = 525).

Overall, 19.1% of the patients with low SES died following an event within 10 years from diagnosis, compared to 15.3% and 13.2% in patients with medium and high SES, respectively (Fig. 2). Median times between first recurrence and death were 2.7 years (IQR:0.9–6.9 years), 2.1 years (IQR:1.0–4.3 years) and 3.3 years (IQR:0.9–5.2 years) for low, medium and high SES, respectively.

### 3.3. Associations between SES, patterns of recurrences and death

**Fig. 3.**
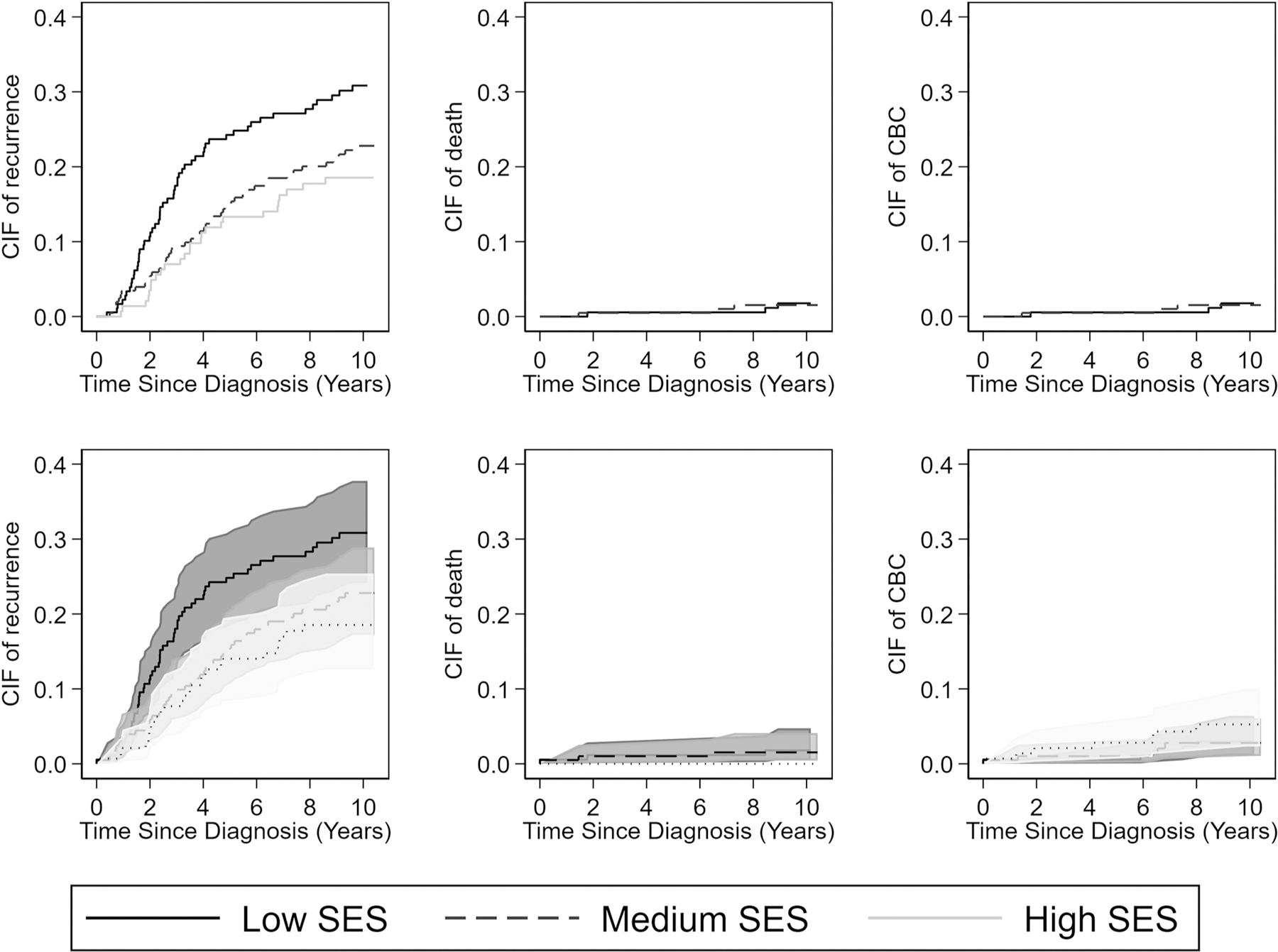
Cumulative incidence function of the 10-year recurrence risk (first event) according to socioeconomic status in women < 40 years with early stage breast cancer (n = 525). Death and CBC as a first event were taken into account as competing event. Abbreviations: CIF = cumulative incidence function, CBC = CBC.

Cumulative incidence functions of any recurrence, death and CBC as first events are illustrated in Fig. 3. Recurrence risks were highest in patients with low SES, lowest in patients with high SES (left panels). Death and CBC as first events were very rare (middle and right panels).

Death occurs more often as subsequent event, following one or more recurrent events (Fig. 2). In a joint model (n = 525, 793 observations) without correction for confounding, high SES was associated with lower recurrence risk compared to low SES (Table 3). After correction for stage, grade and breast cancer subtype, this association was still present (HR:0.30, 95%CI:0.09–1.02), although borderline significant. Note that the estimates are high and confidence intervals around the estimates are very large due to the very low incidence of mortality. The association between 10-year recurrence and excess mortality was positive (ɣ=6.91 (95%CI:1.32–12.51), Table 4), indicating that patients with a higher recurrence risk also have a higher excess mortality risk. After correction for confounding, the variance of the shared random effect (θ) reduced from 17.57 (Tables 3) to 16.46 (Table 4). This is still very high, meaning that despite adjustment on important prognostic factors, unmeasured heterogeneity is likely to be present. Including other factors such as treatment did not alter the associations, but caused a larger uncertainty around the estimates (data not shown).

### 3.4. Sensitivity analysis using the overall mortality hazard

As the expected mortality due to other causes of patients < 40 years is very low, excess mortality estimates are assumed to be similar to overall mortality estimates. We additionally executed the multivariable joint modelling analyses using overall mortality instead of excess mortality as outcome to verify this. Results of this sensitivity analysis were similar to the analyses presented in this paper (Supplementary Table 1).

## 4. Discussion

In this population-based study we showed that high SES is associated with lower risks of recurrence and less subsequent recurrences in young breast cancer patients compared to low SES. This association is independent of stage, breast cancer subtype, grade, treatment and time to recurrence. There was no relationship between SES and excess mortality, which is likely to be partly explained by the low mortality rates in this population. However, we confirmed that there is a positive association between the entire recurrent event process (so all subsequent events) and excess mortality, which implies that the frequently described association between SES and mortality is related to the recurrence pattern. This suggests that other factors such as lifestyle – which is frequently reported to be associated with SES [25] – are less likely to be related to the reported mortality differences. This is confirmed by a recent publication showing that lifestyle only explained a small proportion of the association between SES and mortality [26]. Notably, our study only includes patients< 40 years with no more than 10-year follow-up. Comorbidities as a result of unhealthy lifestyle occur frequently at older age so are unlikely to be present, and therefore unlikely to have played an evident role. The argument that patients of low SES often have an unhealthy lifestyle which increases recurrence risks [27] therefore does not hold in this study.

### 4.1. Potential explanations for differences in recurrence patterns according to SES

A potential argument, which has not been investigated in this study, that may explain the larger number of (subsequent) recurrences in patients of low SES compared to high SES is lower therapy adherence in the first mentioned group. A previous Dutch study showed that there are minimal socioeconomic differences in chemotherapy and endocrine therapy guideline adherence [28], however, especially for endocrine therapy, long-term adherence may still be lower. Other studies showed that differences in breast cancer treatment exist according to SES, with patients of low SES less often receiving axillary surgery and chemotherapy than patients of high SES [29,30]. This is contrasting to our study, in which axillary surgery (including sentinel lymph node procedures) and chemotherapy are not significantly different among the groups. Here, patients of high SES more often received endocrine therapy, but this was correlated with the higher number of hormonal receptor positive patients which was corrected for in the analysis (subtype). Although dated, a study in the Netherlands implied that survival differences among SES were not related to treatment, but that stage at diagnosis largely explains these inequalities [31]. Here, in which we only focused on patients < 40 years – as survival differences according to SES are most pronounced in this group [11,12] – we indeed found significant differences in stage distribution, but contrasting to what is described in literature [3] patients of high SES more often presented with stage II-III disease as compared to low and medium SES. We could not find an explanation for this. Additionally, patients of low SES in our cohort less often received mastectomy with radiotherapy compared to patients of high SES, who more often received postmastectomy radiotherapy. Differences in treatment strategies were reported earlier [32]. However, this was not statistically significant and adding radiotherapy to the multivariable model did not change the results. In our multivariable joint model we still found considerable between-patient variability (θ = 16.46), which indicate the presence of unmeasured factors. One hypothesis that may partly explain the observed recurrence and survival differences is that patients of low SES have prognostically more unfavorable recurrences as compared to patients of high SES. In the low SES group, more patients experienced a (distant) recurrence and subsequently died, compared to medium and high SES. Furthermore, both the median time to a first recurrence and the median time between recurrence and death was lower in patients with low SES compared to medium and high SES. We additionally showed that in the group of low SES more patients had HR-/HER2- disease, which is associated with shorter time to recurrence and unfavorable prognosis compared to the other subtypes [33]. Something not investigated in this study but what might be important is treatment of recurrences. Less optimal treatment in the low socioeconomic group may possibly have led to the shorter time intervals between recurrence and death.

### 4.2. Strengths and limitations

To the best of our knowledge, this is the first population-based study in breast cancer patients < 40 years investigating the relationship between SES and recurrence patterns over 10-year follow-up, rather than mortality only. Our study mainly differs from previously executed studies by jointly modelling recurrence patterns and excess mortality. We specifically aimed to jointly estimate both the recurrence process and mortality, while accounting for the correlation between these two outcomes, in order to get a better understanding of the relationship between SES, recurrence patterns and excess mortality. In a separate study, to complement our results, another approach based on mediation analysis could be conducted, where we would aim to quantify the effect of SES on mortality, potentially mediated by recurrences (indirect effect) [34,35]. The use of the nationwide NCR increases generalizability of our results, and the active follow-up in which all subsequent recurrences were registered provides us with detailed information about prognosis. We expect this active follow-up to be largely complete, as the patterns of recurrence closely resemble the patterns found in other literature. However, in case a patient moves for example to another country, the patient is censored at time of emigration. Any recurrence that occurred after this date is missed. However, as only four patients who were still alive did not have the complete 10 years of follow-up, we potentially only missed recurrences of four patients (spread over the three SES groups). Therefore, we expect this potential limitation to be very minor. Furthermore, as everyone in the Netherlands has equal access to health care and our data managers have access to almost all hospitals in the Netherlands, we do not expect bias in the collection of recurrences according to SES. In our study we used postal code of incidence to determine SES, as we lacked information on education or household income. However, postal codes have been described to be useful markers of SES [36] and have been used in many studies, which allows us to compare our results. The lack of information on ethnicity, comorbidity, performance status, smoking status and BMI can be considered a limitation, as they can all affect the outcomes. For example, it has been described that aromatase inhibitors in obese hormonal receptor positive breast cancer patients may not be as effective as in normal weight women, and in this way relates to higher recurrence risks [37]. Importantly, as we investigated excess mortality, we largely corrected for age-related (including presence of comorbidities) mortality, and in this young population we do not expect much comorbidities. However, people of low SES in general have higher expected mortality than patients of high SES [38], and the unavailability of life tables stratified by SES could have amplified the association between SES and the excess hazard [39]. Our population consisted of patients ≤ 40 years at diagnosis (so with a maximum of 50 years at the end of follow-up) and the expected mortality rates remain very low for those ages. Therefore, the results should not have been affected by the lack of lifetables stratified by SES, as we confirmed with our sensitivity analysis.

### 4.3. Conclusions and future recommendations

High SES is associated with lower recurrence rates, less subsequent recurrent events, and the pattern of recurrence is largely associated with the risk of mortality over 10-year follow-up. Patients of low SES have shorter time intervals between diagnosis and first recurrence, and shorter time intervals between first recurrence and death. Patients of low SES more often have HR-/HER2- disease, suggesting that other breast cancer risk factors play a role. Differences in treatment of recurrences was not assessed in this study and should be subject for further research, to further reduce socioeconomic differences.

## Supplementary Material

1

## Figures and Tables

**Table 1:** Patient, tumor and treatment-related characteristics according to socioeconomic status (n = 525).

Characteristic	Low SES (n = 178)		Medium SES (n = 203)		High SES (n = 144)		p-value*
Median age in years (IQR)	36 (33–38)		36 (33–38)		36 (33–38)		0.873
	n	%	n	%	n	%	
**Lateralization**							
Left	92	51.7%	101	49.8%	70	48.6%	0.854
Right	86	48.3%	102	50.2%	74	51.4%	
**Sublocalization**							
Outer quadrants	86	48.3%	102	50.2%	71	49.3%	0.876
Inner quadrants	26	14.6%	37	18.2%	24	16.7%	
Central parts	10	5.6%	9	4.4%	8	5.6%	
Overlapping lesions	55	30.9%	51	25.1%	37	25.7%	
Unknown	1	0.6%	4	2.0%	4	2.8%	
**Histology**							
Ductal	158	88.8%	177	87.2%	122	84.7%	0.767
Lobular	4	2.2%	9	4.4%	8	5.6%	
Mixed ductal lobular	6	3.4%	8	3.9%	7	4.9%	
Other	10	5.6%	9	4.4%	7	4.9%	
**Differentiation grade**							
Well differentiated	18	10.1%	20	9.9%	21	14.6%	0.305
Moderately differentiated	47	26.4%	67	33.0%	49	34.0%	
Poorly differentiated	101	56.7%	97	47.8%	72	50.0%	
Unknown	12	6.7%	19	9.4%	2	1.4%	
**Multifocality**							
No	134	75.3%	162	79.8%	103	71.5%	0.185
Yes	43	24.2%	36	17.7%	36	25.0%	
Unknown	1	0.6%	5	2.5%	5	3.5%	
**TNM stage**							
I	72	40.5%	85	41.9%	36	25.0%	
II	84	47.2%	89	43.8%	87	60.4%	
III	22	12.4%	29	14.3%	21	14.6%	**0.012**
**Subtype**							
HR+ /HER2−	80	44.9%	89	43.8%	77	53.5%	0.270
HR+ /HER2 +	25	14.0%	31	15.3%	23	16.0%	
HR−/HER2 +	16	9.0%	10	4.9%	7	4.9%	
HR−/HER2−	43	24.2%	46	22.7%	23	16.0%	
Unknown	14	7.9%	27	13.3%	14	9.7%	
**Type of surgery (combined with RT)**							
Lumpectomy with RT	94	52.8%	111	54.7%	80	55.6%	0.811
Mastectomy without RT	61	34.3%	65	32.0%	41	28.5%	
Mastectomy with RT	23	12.9%	27	13.3%	23	16.0%	
**Sentinel node procedure**							
No	76	42.7%	83	40.9%	63	43.8%	
Yes	102	57.3%	120	59.1%	81	56.3%	0.860
**Axillary lymph node dissection**							
No	162	91.0%	178	87.7%	127	88.2%	0.553
Yes	16	9.0%	25	12.3%	17	11.8%	
**Endocrine therapy**							
No	92	61.7%	100	49.3%	55	38.2%	
Yes	86	48.3%	103	50.7%	89	61.8%	**0.039**
**Chemotherapy**							
No	34	19.1%	46	22.7%	22	15.3%	
Yes	144	80.9%	157	77.3%	122	84.7%	0.229
**Targeted therapy**							
No	149	83.7%	167	82.3%	120	83.3%	0.927
Yes	29	16.3%	36	17.7%	24	16.7%	
**Type of hospital**							
Other	162	91.0%	191	94.1%	135	93.8%	0.458
Academic	16	9.0%	12	5.9%	9	6.3%	

Abbreviations: SES = socioeconomic status, n = number, IQR = interquartile range, TNM = tumor, node and metastasis classification system, RT = radiotherapy, ER = oestrogen receptor, PR = progesterone receptor, HER2 = human epidermal growth factor receptor 2.

* A p-value < 0.1 was considered to be statistically significant and indicated in bold. The p-value was calculated using a Chi-squared test (categorical variables) or Mann-Whitney U test (continuous variables) for known values only. In case of an expected frequency < 5 in a cell Fisher’s Exact test was used.

**Table 2:** Number and type of first event within 10-years according to socioeconomic status in breast cancer patients < 40 years (n = 525).

		First event							First or subsequent event					
		
		Low SES (n = 178)		Medium SES (n = 203)		High SES (n = 144)			Low SES (n = 178)		Medium SES (n = 203)		High SES (n = 144)	
**Type of event**	*Total n*	n	%	n	%	n	%	*Total n*	n	%	n	%	n	%
**Any recurrence**	124	53	29.8%	45	22.2%	26	18.1%	124	53	29.8%	45	22.2%	26	18.1%
Local recurrence	29	14	7.9%	9	4.4%	6	4.2%	35	17	9.6%	11	5.4%	7	4.9%
Regional recurrence	18	7	3.9%	7	3.5%	4	2.8%	29	14	7.9%	11	5.4%	4	2.8%
Distant metastasis	77	32	18.0%	29	14.3%	16	11.1%	100	41	23.0%	36	17.7%	23	16.0%
**Contralateral breast cancer, invasive**	14	5	2.8%	4	2.0%	5	3.5%	16	6	3.4%	4	2.0%	6	4.2%
**Contralateral breast cancer, in situ**	4	1	0.6%	1	0.5%	2	1.4%	4	1	0.6%	1	0.5%	2	1.4%
**Death**	6	3	1.7%	3	1.5%	0	0.00%	90	37	20.8%	34	16.8%	19	13.2%

Abbreviations: SES = socioeconomic status, n = number.

* A p-value < 0.1 was considered to be statistically significant and indicated in bold. The p-value was calculated using a Chi-squared test or Fisher’s Exact test In case of an expected frequency < 5 in a cell. In case of 0 observations in a cell the p-value could not be calculated.

**Table 3:** Crude association between socioeconomic status and 10-year rate of recurrence and excess mortality in patients < 40 years in a joint modelling framework (n = 525, 793 observations).

Parameter	Hazard ratio (95% CI)	p-value
**10-year recurrence**		
Low socioeconomic status	reference	
Medium socioeconomic status	0.44 (0.15–1.24)	0.113
High socioeconomic status	0.22 (0.07–0.71)	0.016
**10-year excess mortality**		
Low socioeconomic status	*reference*	
Medium socioeconomic status	0.01 (0.00–9.19)	0.178
High socioeconomic status	0.00 (0.00–2.04)	0.072
	**Coefficient**	**p-value**
θ	17.57	< 0.001
ɣ	6.00	< 0.001

Abbreviations: CI = confidence interval, θ = variance of the random effect, ɣ = scale parameter for the random effect.

**Table 4:** For confounding adjusted association between socioeconomic status and 10-year rate of recurrence and excess mortality in patients < 40 years in a joint modelling framework.

Parameter	Hazard ratio (95%CI)	p-value	Hazard ratio (95%CI)	p-value	Hazard ratio (95%CI)	p-value
	Including stage (n = 525, 793 observations)		Including stage, subtype (n = 470, 709 observations)*		Including stage, subtype, grade (n = 446, 670 observations)* *	
**10-year recurrence**						
Low socioeconomic status	*reference*		*reference*		*reference*	
Medium socioeconomic status	0.44 (0.15–1.24)	0.121	0.44 (0.15–1.31)	0.138	0.52 (0.17–1.60	0.251
High socioeconomic status	0.22 (0.07–0.71)	0.011	0.23 (0.06–0.85)	0.028	0.30 (0.09–1.02)	0.053
Stage I	*reference*		*reference*		*reference*	
Stage II/III	2.08 (0.82–5.30)	0.123	1.97 (0.66–5.89)	0.227	1.90 (0.67–5.34)	0.224
HR+ /HER2− subtype			*reference*		*reference*	
HR+ /HER2 + subtype			0.37 (0.08–1.79)	0.216	0.39 (0.10–1.58)	0.186
HR−/HER2 + subtype			1.37 (0.21–8.88)	0.741	0.89 (0.12–6.54)	0.908
HR−/HER2− subtype			1.26 (0.39–4.06)	0.693	0.89 (0.23–3.39)	0.869
Grade 3 (poorly differentiated)					*reference*	0.174
Grade II/II (well/moderately differentiated)					0.46 (0.15–1.41)	
**10-year excess mortality**						
Low socioeconomic status	*reference*	*reference*		*reference*		
Medium socioeconomic status	0.01 (0.00–9.96)	0.187	0.01 (0.00–30.16)	0.249	0.03 (0.00–130.7)	0.400
High socioeconomic status	0.00 (0.00–1.53)	0.062	0.00 (0.00–16.96)	0.146	0.00 (0.00–43.5)	0.220
Stage I	*reference*		*reference*		*reference*	
Stage II/III	89.24 (0.14–5.8 *10^4^)	0.174	192.9 (0.03–1.12 *10^6^)	0.234	150.7 (0.04–6.41 *10^5^)	0.239
HR+ /HER2− subtype			*reference*		*reference*	
HR+ /HER2 + subtype			0.00 (0.00–67.30)	0.193	0.00 (0.00–42.1)	0.181
HR−/HER2 + subtype			2.51 (0.00–7.29 *10^5^)	0.886	0.05 (0.00–7.1 *10^4^	0.683
HR−/HER2− subtype			26.97 (0.01–1.19 *10^5^)	0.441	2.90 (0.00–3.59 *10^4^)	0.825
Grade 3 (poorly differentiated)					*reference*	
Grade II/II (well/moderately differentiated)					0.00 (0.00–16.9)	0.164
	**Coefficient (95%CI)**	**p-value**	**Coefficient**	**p-value**	**Coefficient (95%CI)**	**p-value**
θ	17.20 (11.79–22.62)	< 0.001	16.79 (10.96–22.61)	< 0.001	16.46 (10.65–22.28)	< 0.001
ɣ	6.02 (2.33–9.71)	0.001	6.65 (1.54–11.75)	0.011	6.91 (1.32–12.51)	0.001

Abbreviations: CI = confidence interval, HR = hormonal receptors, HER2 = human epidermal growth factor receptor 2, θ = variance of the random effect, ɣ = scale parameter for the random effect. In the multivariable models * 55 patients and * *79 patients were excluded from the multivariable analysis due to missing values (≈15%). Due to statistical complexities it was decided not to impute these missing values.
