# Supplementary material for: Socioeconomic status and its relation with breast cancer recurrence and survival in young women in the Netherlands
Source: Cancer Epidemiol. Author manuscript; Available in PMC 2022 Aug 29. (PMC9422085; doi:10.1016/j.canep.2022.102118)
Supplement: 1 [file NIHMS1821269-supplement-1.pdf]

Supplementary material

Supplementary Table 1. For confounding adjusted association between socioeconomic status and 10-year rate of recurrence and overall mortality in patients <40 years in a joint modelling framework

**Parameter**	**Hazard ratio (95%CI)**	**p-value**	**Hazard ratio (95%CI)**	**p-value**	**Hazard ratio (95%CI)**	**p-value**
	**Including stage (n=525, 793 observations)**		**Including stage, subtype (n=470, 709 observations)***		**Including stage, subtype, grade (n=446, 670 observations)****	
**10-year recurrence**						
Low socioeconomic status	*reference*		*reference*		*reference*	
Medium socioeconomic status	0.44 (0.15-1.24)	0.120	0.43 (0.15-1.30)	0.136	0.52 (0.17-1.60)	0.253
High socioeconomic status	0.22 (0.07-0.70)	0.011	0.23 (0.06-0.83)	0.026	0.30 (0.09-1.02)	0.055
Stage I	*reference*		*reference*		*reference*	
Stage II/III	2.08 (0.82-5.28)	0.123	1.97 (0.67-5.85)	0.220	1.90 (0.68-5.33)	0.228
HR+/HER2- subtype			*reference*		*reference*	
HR+/HER2+ subtype			0.37 (0.08-1.73)	0.207	0.39 (0.10-1.57)	0.183
HR-/HER2+ subtype			1.36 (0.21-8.78)	0.745	0.89 (0.12-6.52)	0.909
HR-/HER2- subtype			1.27 (0.40-4.06)	0.688	0.90 (0.24-3.40)	0.875
Grade 3 (poorly differentiated)					*reference*	
Grade II/II (well/moderately differentiated)					0.00 (0.00-19.79)	0.173
**10-year excess mortality**						
Low socioeconomic status	*reference*		*reference*		*reference*	
Medium socioeconomic status	0.01 (0.00-10.52)	0.188	0.01 (0.00-36.02)	0.253	0.02 (0.00-157.42)	0.403
High socioeconomic status	0.00 (0.00-1.55)	0.063	0.00 (0.00-19.62)	0.149	0.00 (0.00-52.96)	0.226
Stage I	*reference*		*Reference*		*reference*	
Stage II/III	99.98 (0.13-7.84*10^{4})	0.176	242.26 (0.03-1.99*10^{6})	0.234	170.03 (0.03-9.35*10^{5})	0.243
HR+/HER2- subtype			*reference*		*reference*	
HR+/HER2+ subtype			0.00 (0.00-75.11)	0.192	0.00 (0.00-50.25)	0.183
HR-/HER2+ subtype			2.52 (0.00-1.11*10^{6})	0.889	0.05 (2.44*10^{8}-9.88*10^{4})	0.684
HR-/HER2- subtype			31.25 (0.01-1.93*10^{5})	0.440	3.10 (1.87*10^{4}-5.15*10^{4})	0.819

Grade 3 (poorly differentiated)					*reference*	
Grade II/II (well/moderately differentiated)					0.00 (0.00-19.79)	0.167
	**Coefficient (95%CI)**	**p-value**	**Coefficient**	**p-value**	**Coefficient (95%CI)**	**p-value**
θ	17.19 (11.80-22.58)	<0.001	16.78 (11.00-22.56)	<0.001	16.48 (10.68-22.28)	<0.001
ɣ	6.17 (2.33-10.01)	0.002	6.88 (1.35-12.41)	0.015	7.13 (1.21-13.05)	0.019

Abbreviations: CI = confidence interval, HR = hormonal receptors, HER2 = human epidermal growth factor receptor 2, θ = variance of the random effect, ɣ = scale parameter for the random effect. In the multivariable models *55 patients and **79 patients were excluded from the multivariable analysis due to missing values (≈15%). Due to statistical complexities it was decided not to impute these missing values.

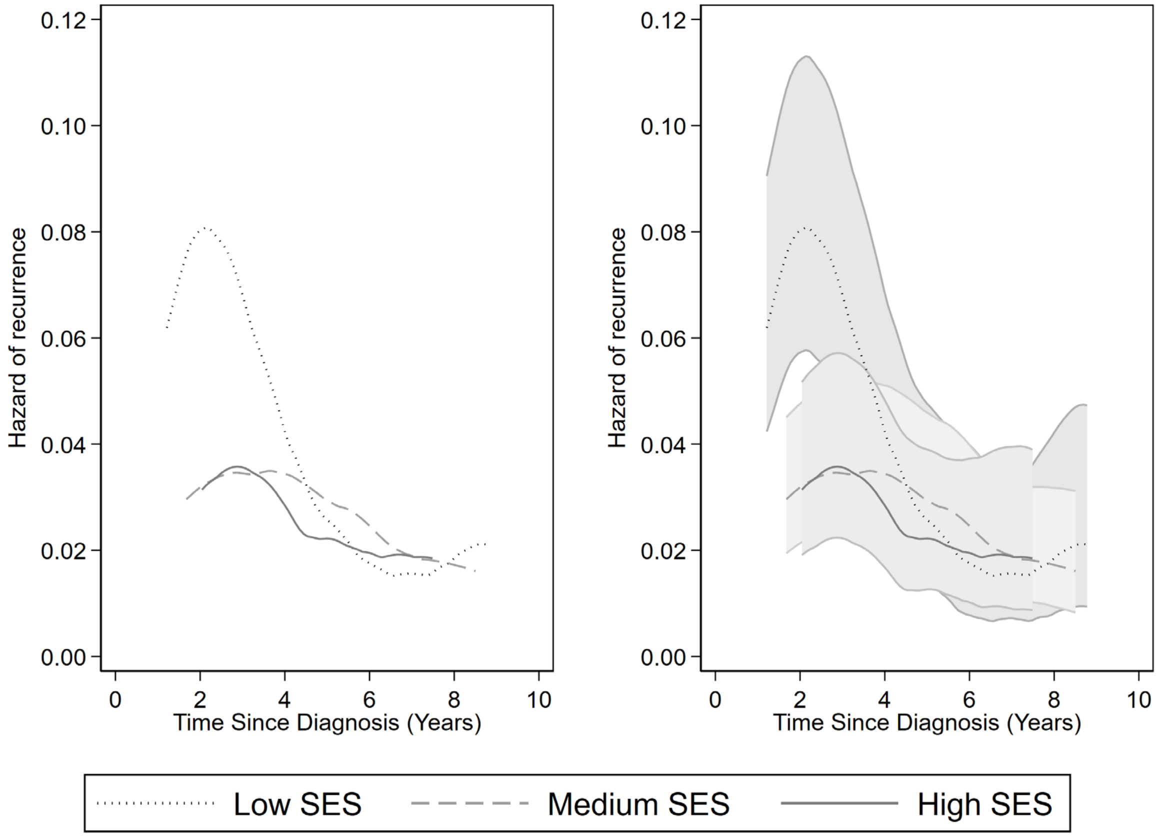

Supplementary Figure 1. Hazards of recurrence per socioeconomic status. Left panel = without confidence intervals, right panel = with 95% confidence intervals.

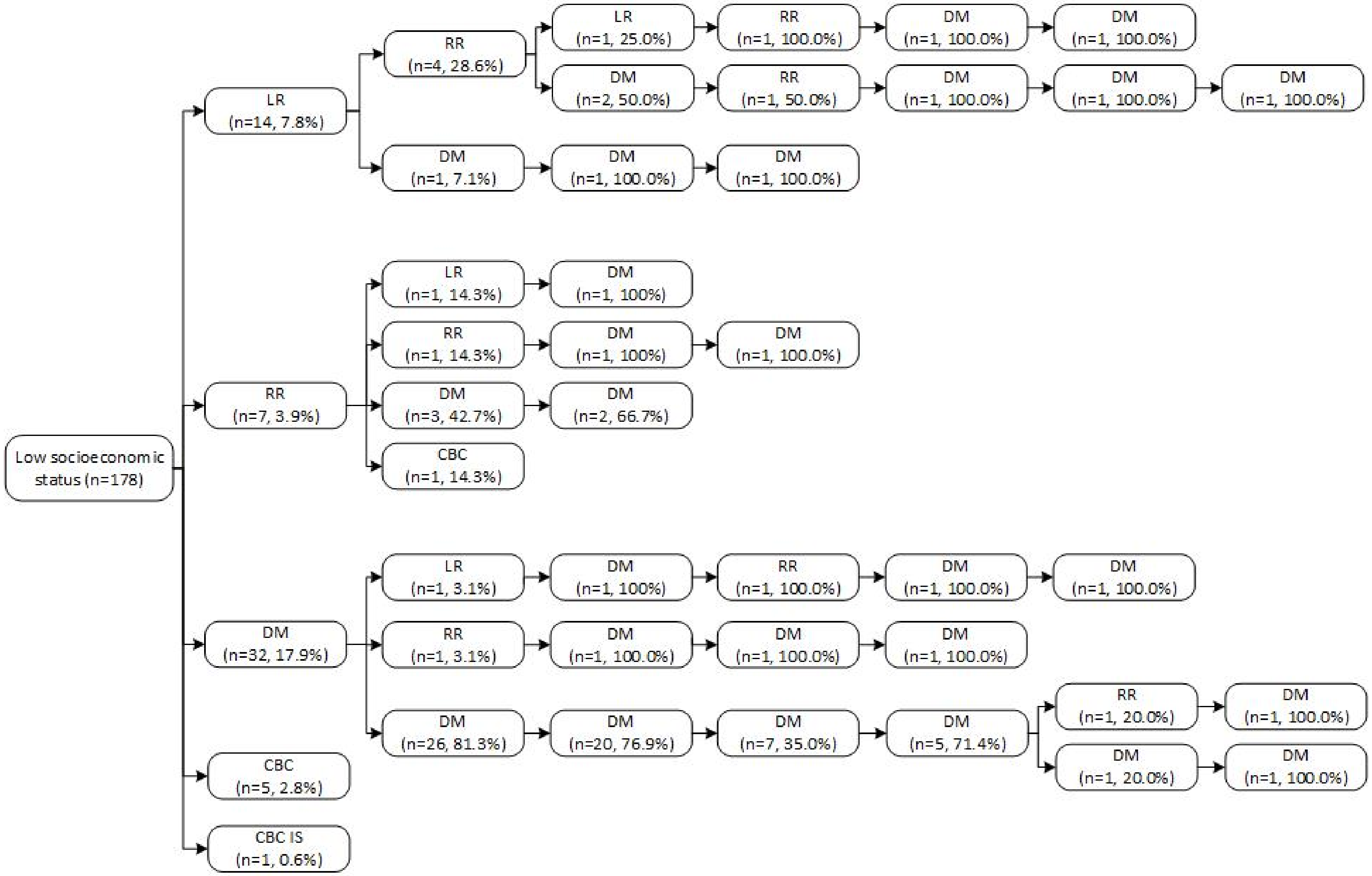

Supplementary Figure 2. Patterns of recurrences in women <40 years with low socioeconomic status (n=178). Abbreviations: LR=local recurrence, RR=regional recurrence, DM=distant metastasis, CBC=contralateral breast cancer, IS = In situ.

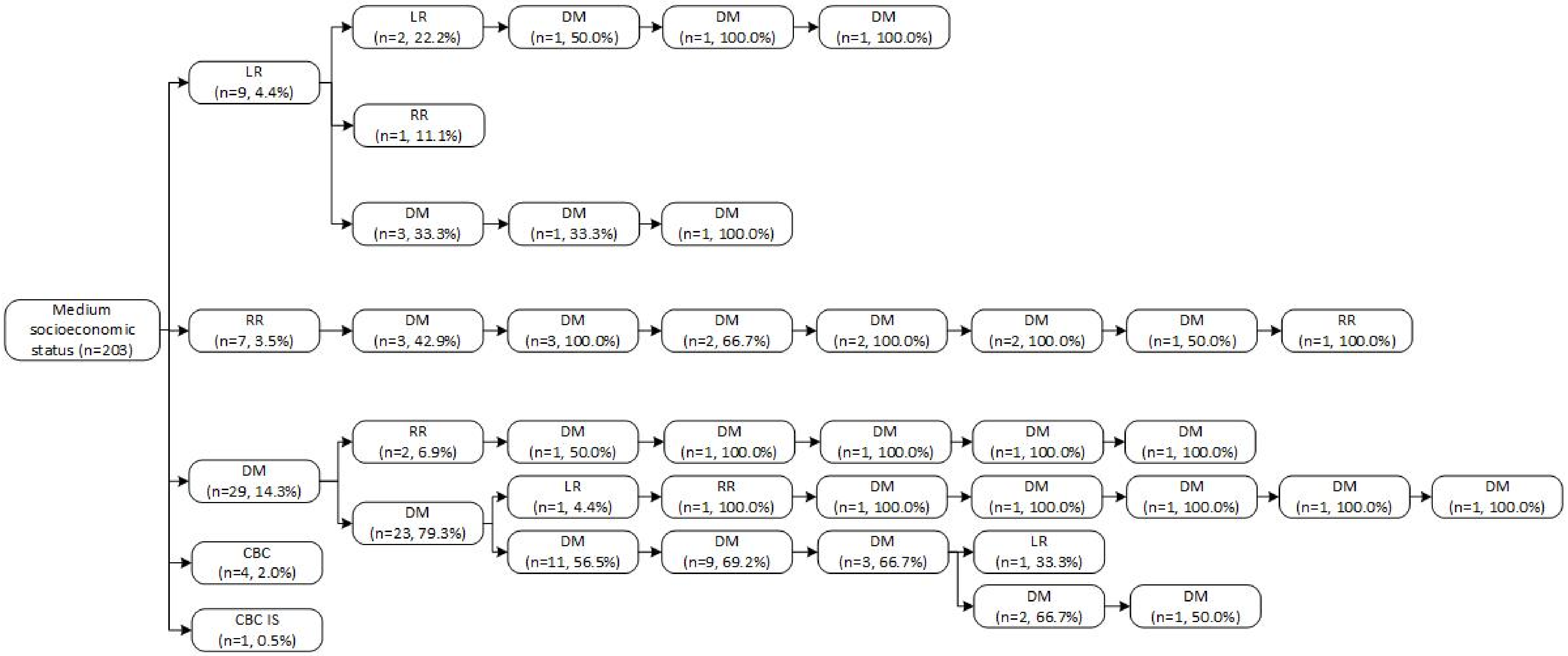

Supplementary Figure 3. Patterns of recurrences in women <40 years with medium socioeconomic status (n=203). Abbreviations: LR=local recurrence, RR=regional recurrence, DM=distant metastasis, CBC=contralateral breast cancer, IS = In situ.

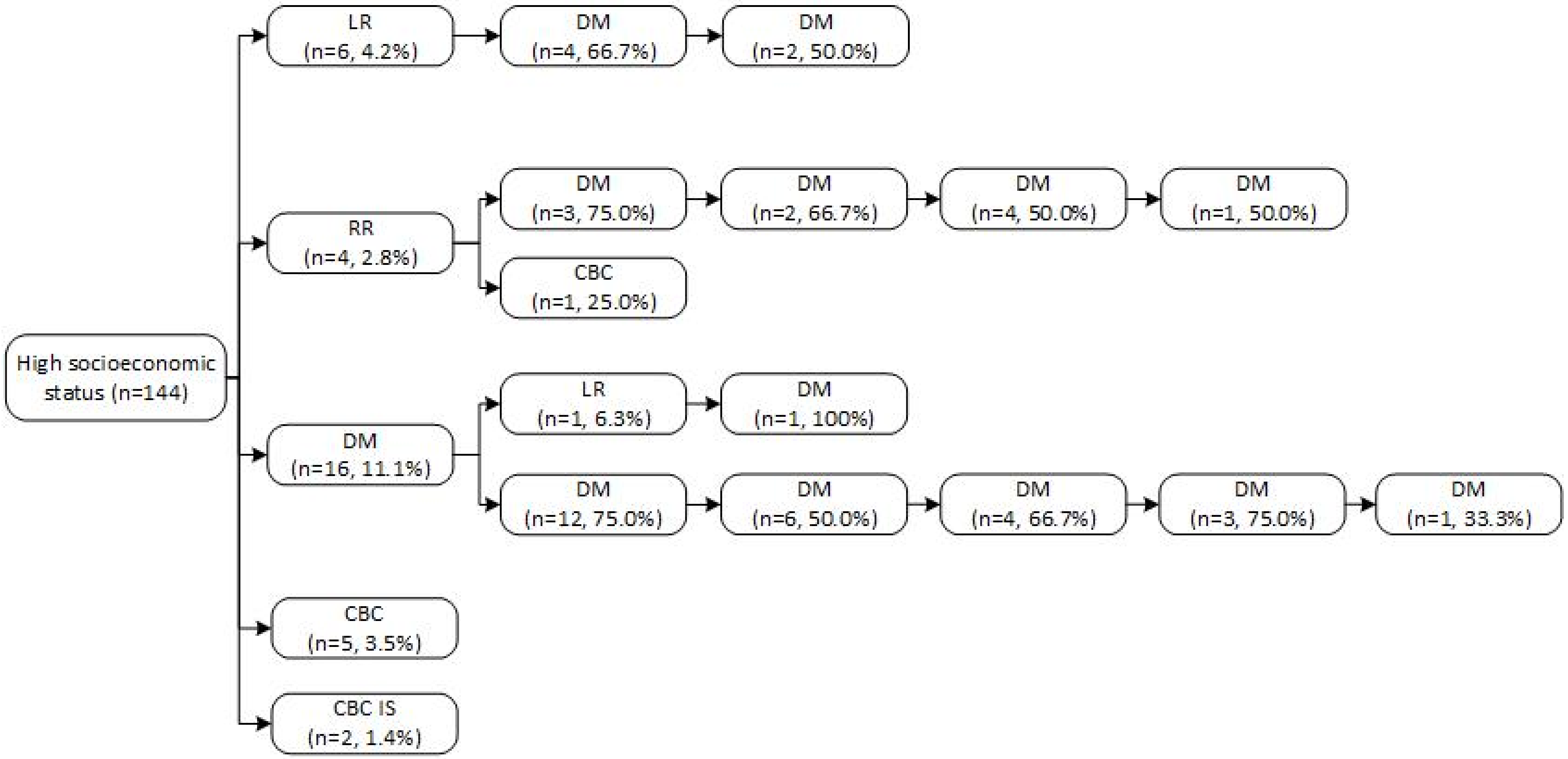

Supplementary Figure 4. Patterns of recurrences in women <40 years with high socioeconomic status (n=144). Abbreviations: LR=local recurrence, RR=regional recurrence, DM=distant metastasis, CBC=contralateral breast cancer, IS = In situ.
